# The Chemical Compositions of the Volatile Oils of Garlic (*Allium sativum*) and Wild Garlic (*Allium vineale*)

**DOI:** 10.3390/foods6080063

**Published:** 2017-08-05

**Authors:** Prabodh Satyal, Jonathan D. Craft, Noura S. Dosoky, William N. Setzer

**Affiliations:** Department of Chemistry, University of Alabama in Huntsville, Huntsville, AL 35899, USA; prabodhsatyal@gmail.com (P.S.); craftjd@gmail.com (J.D.C.); nouradosoky@yahoo.com (N.S.D.)

**Keywords:** *Allium sativum*, *Allium vineale*, essential oil composition, allyl polysulfides, cluster analysis

## Abstract

Garlic, *Allium* *sativum*, is broadly used around the world for its numerous culinary and medicinal uses. Wild garlic, *Allium vineale*, has been used as a substitute for garlic, both in food as well as in herbal medicine. The present study investigated the chemical compositions of *A. sativum* and *A. vineale* essential oils. The essential oils from the bulbs of *A. sativum*, cultivated in Spain, were obtained by three different methods: laboratory hydrodistillation, industrial hydrodistillation, and industrial steam distillation. The essential oils of wild-growing *A. vineale* from north Alabama were obtained by hydrodistillation. The resulting essential oils were analyzed by gas chromatography-flame ionization detection (GC-FID) and gas chromatography-mass spectrometry (GC-MS). Both *A. sativum* and *A. vineale* oils were dominated by allyl polysulfides. There were minor quantitative differences between the *A. sativum* oils owing to the distillation methods employed, as well as differences from previously reported garlic oils from other geographical locations. *Allium vineale* oil showed a qualitative similarity to *Allium ursinum* essential oil. The compositions of garlic and wild garlic are consistent with their use as flavoring agents in foods as well as their uses as herbal medicines. However, quantitative differences are likely to affect the flavor and bioactivity profiles of these *Allium* species.

## 1. Introduction

Garlic (*Allium sativum* L., Amaryllidaceae) likely originated in Central Asia [1]. The plant has been used as a flavoring agent and a traditional medicine since antiquity, and is now cultivated worldwide [1,2]. *Allium vineale* L. (wild garlic, crow garlic) is native to Great Britain, most of Europe, North Africa, and the Middle East. The plant has been introduced to North America, Australia, and New Zealand [3].

*Allium sativum* has been used as a diaphoretic, diuretic, expectorant, and stimulant [4]. Extracts of *A. sativum* have shown broad-spectrum antibacterial [5] and antifungal [6] activity and the plant has been used to treat tuberculosis, coughs, and colds [7]. Garlic preparations have demonstrated hypotensive activity in moderately hypertensive subjects, and garlic-based phytotherapeutic products are used in France for minor vascular disorders [8]. There is an inverse correlation between regular consumption of garlic and stomach cancer frequency [8], but there seems to be no correlation between garlic consumption and other cancers. Garlic has been used in food preparation not only for its flavor, but also as a digestive aid [4]. *Allium vineale* has been used as a substitute for *A. sativum* in cooking; the bulb is used as a flavoring agent and the leaves as an addition to salad [9,10]. Cherokee Native Americans used both *A. vineale* and *A. sativum* as carminatives, diuretics, and expectorants [11,12].

Although there have been numerous investigations on the phytochemistry of garlic (*A. sativum*) [1,13,14], the chemistry of wild garlic (*A. vineale*) has not been investigated, and because of the history of the uses of *Allium* species as both condiments and phytopharmaceuticals, we have investigated the essential oil compositions of *A. sativum* from Spain, obtained by different isolation methods, and *A. vineale* growing wild in north Alabama, USA.

## 2. Materials and Methods

### 2.1. Plant Material

#### 2.1.1. Allium sativum

Bulbs of *Allium sativum* were collected from a field in Las Pedroñeras, Spain (39°26′59″ N, 2°40′23″ W, 745 m elevation), in December 2015. Garlic bulbs were finely chopped, and were subjected to three different distillation methods: laboratory hydrodistillation using a Clevenger apparatus for 3 h, industrial hydrodistillation for 4 h, and industrial steam distillation for 5 h. Pale yellow essential oils were obtained in 0.2%, 0.22% and 0.18% yields, respectively. The obtained essential oils and hydrosol were separated by decantation; remaining water was removed from the essential oils with sodium chloride. The collected essential oil samples were stored under refrigeration (−4 °C) until analysis.

#### 2.1.2. Allium vineale

Four different samples of *Allium vineale* were collected from a field in Huntsville, Alabama (34°38′46″ N, 86°33′27″ W, 191 m elevation) on 10 April 2017, 8 a.m. Each sample was cleaned of debris, the entire plant (leaves and bulbs) chopped, and hydrodistilled using a Likens-Nickerson apparatus for 4 h with continuous extraction with dichloromethane (CH_2_Cl_2_). Evaporation of the dichloromethane yielded pale yellow essential oils with an extremely pungent odor (Table 1).

### 2.2. Gas Chromatography-Mass Spectrometry (GC-MS)

GC-MS characterization of *A. sativum* oils was carried out as previously described using a Shimadzu GCMS-QP2010 Ultra (Shimadzu Scientific Instruments, Columbia, MD, USA) [15,16]. This instrument was operated in the electron impact (EI) mode set at electron energy 70 eV with a scan range of 40–400 amu, a scan rate of 3.0 scans per second, and with GC-MS solution software. A ZB-5 fused silica capillary column (Phenomenex, Torrance, CA, USA), 30 m length × 0.25 mm inner diameter, with a (5% phenyl)-polymethylsiloxane stationary phase and a film thickness of 0.25 μm was used as the GC column. Helium was used as the carrier gas and the pressure was set at 551.6 kPa with a flow rate of 1.37 mL/min on the column head. The temperature of the injector was set at 250 °C and the temperature of the ion source was set at 200 °C. The temperature of the GC oven was programmed to be 50 °C initially and was programmed to increase at a rate of 2 °C/min to a final temperature of 260 °C. The samples were prepared with CH_2_Cl_2_ in a 5% *w*/*v* solution. Then, 0.1 µL of the solutions were injected into the instrument with a split ratio of 30:1.

GC-MS analysis of *A. vineale* oils was carried out as previously described [17]: Agilent 6890 GC (Agilent Technologies, Santa Clara, CA, USA), Agilent 5973 mass selective detector (Agilent Technologies), EI mode (70 eV), 40–400 mass scan range, 3.99 scans/s scan rate, and operated through an Agilent ChemStation data system (G1701CA, Agilent Technologies); HP-5ms capillary column (30 m length × 0.25 mm inner diameter × 0.25 μm film thickness), helium carrier gas, head pressure (92.4 kPa), flow rate (1.5 mL/min); oven temperature program (60 °C initial temperature, which was held for 5 min, temperature increased at a rate of 3 °C/min up to 280 °C), inlet temperature (250 °C), interface temperature (280 °C). *Allium vineale* solutions (1 μL of 1% in CH_2_Cl_2_) were injected using a splitless mode.

The retention indices were determined by reference to a homologous series of *n*-alkanes. The components of each essential oil sample were identified based on their retention indices and mass spectral fragmentation patterns compared to reference literature [18,19,20,21,22] and our in-house library.

### 2.3. Semi-Quantitative Gas Chromatography

Semi-quantitative GC was performed with an Agilent 6890 GC with Agilent FID (flame ionization detector) (Agilent Technologies), HP-5ms column (30 m length × 0.25 mm inner diameter × 0.25 μm film thickness), He carrier gas, head pressure (144.1 kPa), flow rate (2.0 mL/min); oven temperature program (as above). The percent compositions of the essential oils were determined from raw peak area percentages without standardization.

### 2.4. Hierarchical Cluster Analysis

The chemical compositions of *A. sativum* from this current study along with garlic oil compositions from previously published works (hydrodistillations and steam distillations only) [6,23,24,25,26,27,28,29,30] were used as operational taxonomic units (OTUs). The percentages of the major sulfur-containing compounds (diallyl sulfide, allyl methyl disulfide, dimethyl trisulfide, diallyl disulfide, allyl (*Z*)-1-propenyl disulfide, allyl (*E*)-1-propenyl disulfide, allyl methyl trisulfide, 2-vinyl-4*H*-1,3-dithiine, diallyl trisulfide, and diallyl tetrasulfide) were used to evaluate the chemical similarities and differences between the garlic oil samples by agglomerative hierarchical cluster (AHC) analysis using the XLSTAT software, version 2015.4.01 (Addinsoft™, New York, NY, USA). Pearson correlation was used to evaluate similarity and clusters were defined by the unweighted pair-group method with arithmetic averaging (UPGMA). 

## 3. Results and Discussion

### 3.1. *Allium sativum*

**Figure 1:**
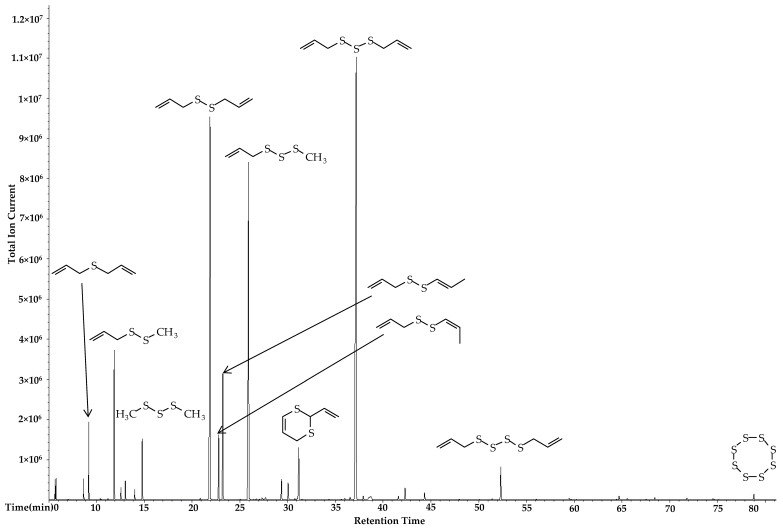
Chromatogram of *Allium sativum* essential oil from Clevenger distillation, including major sulfur-containing compounds.

The garlic (*A. sativum*) essential oils from Spain, obtained using three different distillation methods (Clevenger laboratory hydrodistillation, industrial steam distillation, and industrial hydrodistillation) were characterized by GC-MS (Table 2). The oils were dominated by allyl polysulfides, including diallyl sulfide (1.9–9.5%), diallyl disulfide (20.8–27.9%), diallyl trisulfide (16.8–33.4%), allyl methyl disulfide (4.4–8.3%), and allyl methyl trisulfide (14.5–19.2%). The major components of *A. sativum* essential oil extracted by Clevenger-type laboratory distillation were diallyl trisulfide (allitridin) (33.4%), diallyl disulfide (20.8%), allyl methyl trisulfide (19.2%), allyl (*E*)-1-propenyl disulfide (5.2%), and allyl methyl disulfide (4.4%) (see Figure 1), whereas the main constituents in the oil extracted by industrial hydrodistillation were diallyl trisulfide (31.2%), diallyl disulfide (25.9%), allyl methyl trisulfide (14.5%), allyl methyl disulfide (5.2%), allyl (*E*)-1-propenyl disulfide (4.6%) and diallyl sulfide (3.4%). Thus, the two hydrodistillation methods yielded very similar results. Extraction by industrial steam distillation, on the other hand, resulted in the identification of diallyl disulfide (27.9%), allyl methyl trisulfide (17.7%), diallyl trisulfide (16.8%), diallyl sulfide (9.5%), allyl methyl disulfide (8.3%), and allyl (*E*)-1-propenyl disulfide (3.7%) as the major components. Thus, the steam distillation gave somewhat increased yields of diallyl sulfide and diallyl disulfide with a concomitant decrease in diallyl trisulfide and diallyl tetrasulfide yields. These differences are small, however; the three distillation methods gave comparable results overall.

The oil compositions from this study show quantitative similarities and differences from previously published reports on garlic oil [6,23,24,25,26,27,28,29,30]. Egyptian garlic essential oil extracted by hydrodistillation had diallyl disulfide (25.2%), allyl methyl trisulfide (23.8%) and diallyl trisulfide (21.1%) as the major constituents [29]. The major components of Serbian garlic essential oil obtained by hydrodistillation were diallyl trisulfide (33.6%), diallyl disulfide (28.1%), and allyl methyl trisulfide (17.8%) [26]. Diallyl disulfide (49.1%) and diallyl trisulfide (30.4%) were the main components of Tunisian garlic essential oil obtained by hydrodistillation [31]. The profile identified in this study was also different from French garlic oil presented by Mnayer et al. [27] in which the major components were diallyl disulfide (37.9%), diallyl trisulfide (28.1%), allyl methyl trisulfide (7.3%), diallyl sulfide (6.6%), diallyl tetrasulfide (4.1%) and allyl methyl disulfide (3.7%). Douiri et al. [23] showed that *A. sativum* essential oil obtained by Clevenger hydrodistillation was dominated by diallyl trisulfide (46.5%) followed by diallyl disulfide (16.0%), allyl methyl trisulfide (10.9%) and diallyl disulfide (7.2%). Similarly, Rao and co-workers have analyzed six geographical varieties of essential oils obtained by steam distillation of fresh garlic grown in India. These investigators found diallyl disulfide (27.1–46.8%) and diallyl trisulfide (19.9–34.1%) to be the dominant components, followed by allyl methyl trisulfide (8.3–18.2%), and allyl methyl disulfide (4.4–12.0%) [28]. Commercial Chinese garlic oil has shown abundant diallyl disulfide (45.1–63.2%), diallyl trisulfide (18.5–23.4%), diallyl sulfide (4.5–11.4%), and diallyl tetrasulfide (6.3–10.5%) (unpublished results from our laboratories). Kimbaris and co-workers obtained garlic oil from Greece (Likens-Nickerson hydrodistillation-extraction) and found diallyl disulfide (23.1–28.4%), diallyl trisulfide (18.2–22.1%), allyl methyl trisulfide (16.3–17.5%), and allyl methyl disulfide (8.5–11.2%) [25].

**Figure 2:**
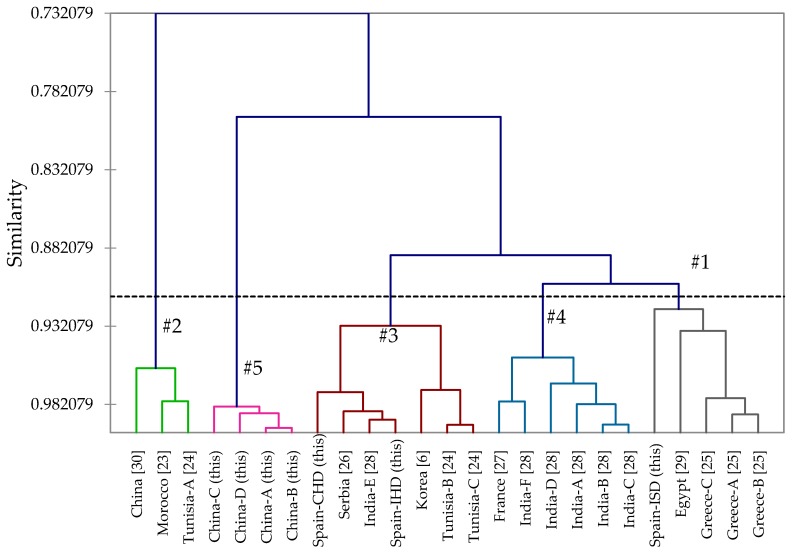
Dendrogram obtained from the agglomerative hierarchical cluster analysis of 25 *Allium sativum* essential oil compositions. Individual clusters are highlighted by different colored lines and numbers (#1–#5). The letters A–F refer to different essential oil samples from the same country of origin. CHD = Clevenger hydrodistillation; IHD = industrial hydrodistillation; ISD = industrial steam distillation.

A hierarchical cluster analysis of garlic oils from this work and reported in the literature has been carried out (Figure 2). The cluster analysis revealed greater than 70% similarity between all oils, but five distinct clusters with greater than 90% similarity for each cluster can be defined based on the relative concentrations of sulfur-containing compounds: Cluster #1 (diallyl disulfide > diallyl trisulfide ≈ allyl methyl trisulfide > allyl methyl sulfide), Cluster #2 (diallyl trisulfide ≫ diallyl disulfide ≫ allyl methyl trisulfide ≈ diallyl tetrasulfide), Cluster #3 (diallyl trisulfide > diallyl disulfide > allyl methyl sulfide), Cluster #4 (diallyl disulfide > diallyl trisulfide > allyl methyl trisulfide > allyl methyl disulfide), and Cluster #5 (diallyl disulfide ≫ diallyl trisulfide ≫ diallyl tetrasulfide ≈ diallyl sulfide). Both of the hydrodistilled samples from Spain in this work fall into Cluster #3, while the steam-distilled sample falls into Cluster #1. Five of the Indian garlic varieties [28] (Rajkot, Gondal, Jamnagar, Junagadh, and Gujarat) are in Cluster #4, while the Amreli variety is in Cluster #3. Four different commercial garlic oil samples from China (unpublished data from our laboratories) form Cluster #5.

### 3.2. *Allium vineale*

**Figure 3:**
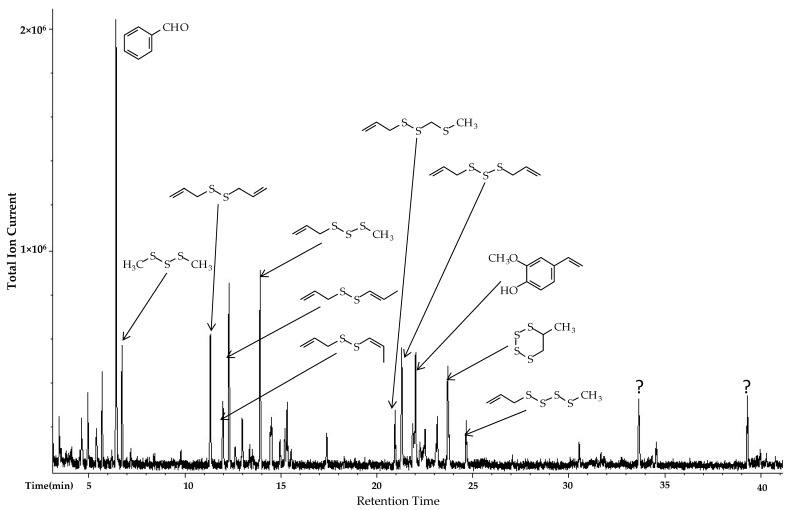
Chromatogram of *Allium vineale* essential oil sample #4 showing major components. ? = unidentified component.

The gas chromatographic analysis of *A. vineale* essential oils is summarized in Table 3. A representative chromatogram (sample #4) is shown in Figure 3. The major components in the essential oils were sulfur-containing compounds allyl methyl trisulfide (7.9–13.2%), allyl (*E*)-1-propenyl disulfide (7.9–12.5%), dimethyl trisulfide (4.3–17.4%), diallyl disulfide (4.4–12.2%), diallyl trisulfide (2.8–10.5%), and methyl (*E*)-1-propenyl disulfide (2.6–12.5%). The high proportion of sulfur-containing components (74.9–91.6%) accounts for the very pungent aroma of the oils.

In contrast to wild garlic (*A. vineale*), garlic (*A. sativum*) essential oils tend to be very rich in allyl polysulfides, especially diallyl disulfide, allyl methyl trisulfide, diallyl trisulfide, and diallyl tetrasulfide (see above) [6,25,27]. The compositions of *A. vineale* are similar to those reported for *Allium ursinum* (broad-leaved garlic, bear’s garlic, wild garlic) volatile oils, which showed allyl methyl disulfide (13.0–18.9%), methyl (*E*)-1-propenyl disulfide (3.4–6.2%), dimethyl trisulfide (3.5–7.5%), diallyl disulfide (16.2–19.9%), allyl (*E*)-1-propenyl disulfide (7.5–10.2%), and allyl methyl trisulfide (12.6–15.0%) [32]. Both *A. vineale* and *A. ursinum* have qualitative similarities in composition to *A. sativum*, which no doubt accounts for the similar uses of these two “wild garlic” species.

The medicinal properties of garlic have been attributed to the abundance of sulfur-containing compounds. These compounds have also shown antifungal [33,34], antibacterial [27,35,36], acaricidal [37], antiparasitic [38,39], nematicidal [40], antiviral [29], and insecticidal [23,30,41,42] properties. Diallyl disulfide and dipropyl disulfide have hypoglycemic [43] and hypolipidemic actions [44]. Diallyl trisulfide and diallyl disulfide, which are allicin-derivative products, have been shown to activate antioxidant enzymes [45,46] and to possess antimicrobial activity [47,48].

## 4. Conclusions

The essential oils of garlic and wild garlic are shown to be dominated by sulfur-containing compounds, particularly allyl polysulfides. Garlic oils from various geographical locations have shown qualitative similarities, but quantitative differences in the concentrations of organosulfur compounds, and are likely to affect both the medicinal and the organoleptic properties of the garlic. Wild garlic is qualitatively similar in composition to garlic, but there are some key differences: diallyl disulfide and diallyl trisulfide concentrations are higher in garlic than in wild garlic, while allyl 1-propenyl disulfide and dimethyl trisulfide concentrations are higher in wild garlic than in garlic.

## Figures and Tables

**Table 1:** Essential oil yields of *Allium vineale*.

Sample	#1 ^a^	#2	#3	#4
Mass of plant material (g)	94.04	123.29	98.20	72.35
Mass of essential oil (mg)	87.2	258.5	210.5	25.3
Essential oil yield	0.0927%	0.2097%	0.2144%	0.0350%

^a^ #1, #2, #3, and #4 are different essential oil samples.

**Table 2:** Essential oil compositions of garlic (*Allium sativum*) obtained by different distillation methods.

RI ^a^	Compound	Percent Composition		
		Clevenger-Type Hydrodistillation	Industrial Steam Distillation	Industrial Hydrodistillation
739	Dimethyl disulfide	0.4	1.4	- ^b^
741	2-Methyl-4-pentenal	tr ^c^	0.1	-
743	2-Methylene-4-pentenal	0.4	-	-
787	3-Methylthiophene	-	0.1	-
801	Hexanal	tr	0.1	-
842	1,2-Dithiolane	0.5	0.3	0.4
855	Diallyl sulfide	1.9	9.5	3.4
870	Allyl propyl sulfide	-	0.1	-
886	Allyl (*Z*)-1-propenyl sulfide	tr	tr	tr
889	Allyl (*E*)-1-propenyl sulfide	tr	-	-
904	3,4-Dimethylthiophene	tr	0.2	0.1
916	Allyl methyl disulfide	4.4	8.3	5.2
928	Methyl (*Z*)-1-propenyl disulfide	0.4	0.4	0.4
936	Methyl (*E*)-1-propenyl disulfide	0.6	0.7	0.6
953	1,2-Dithiolene	0.3	0.1	0.4
968	Dimethyl trisulfide	2.0	2.9	1.3
1080	Diallyl disulfide	20.8	27.9	25.9
1093	Allyl (*Z*)-1-propenyl disulfide	2.6	2.2	2.6
1100	Allyl (*E*)-1-propenyl disulfide	5.2	3.7	4.6
1138	Allyl methyl trisulfide	19.2	17.7	14.5
1149	Methyl propyl trisulfide	-	-	tr
1153	4-Methyl-1,2,3-trithiolane	tr	1.2	0.5
1159	Methyl (*Z*)-1-propenyl trisulfide	0.1	-	0.1
1164	Methyl (*E*)-1-propenyl trisulfide	0.1	-	0.1
1188	3-Vinyl-4*H*-1,2-dithiine	0.9	0.8	0.6
1198	1,2,3-Trithia-4-cyclohexene	0.7	0.4	0.6
1208	Allicin	tr	-	tr
1214	2-Vinyl-4*H*-1,3-dithiine	2.5	1.8	2.0
1292	Methyl (methylsulfinyl)methyl sulfide ^d^	0.1	0.1	0.1
1301	Diallyl trisulfide	33.4	16.8	31.2
1313	Allyl propyl trisulfide	0.2	0.3	0.2
1325	Allyl (*E*)-1-propenyl trisulfide	-	-	0.4
1369	5-Methyl-1,2,3,4-tetrathiane	0.2	0.4	0.6
1379	Unidentified ^e^	0.5	0.7	0.7
1411	1,4-Dihydro-2,3-benzoxathiin 3-oxide	0.4	0.2	0.2
1443	[(*E*)-1-Propenyl] 2-thiopent-3-yl disulfide ^d^	-	0.2	-
1540	Diallyl tetrasulfide	1.5	1.0	2.2
1591	Propyl 4-thiohept-2-en-5-yl disulfide ^d^	-	0.2	-
1646	4-Methyl-1,2,3,5,6-pentathiepane ^c^	-	0.1	0.2
2041	Cyclooctasulfur	0.3	0.1	0.4
	Total Identified	99.2	98.9	99.0
	Sulfur-containing	99.3	99.4	99.5

^a^ RI = Retention index determined with respect to a homologous series of *n*-alkanes on an ZB-5 column. ^b^ - = not detected. ^c^ tr = trace (<0.05%). ^d^ Identification based on MS only. ^e^ MS (m/z): 210 (1%), 184 (3%), 158 (9%), 146 (5%), 120 (32%), 105 (10%), 79 (36%), 64 (60%), 45 (41%), 41 (100%).

**Table 3:** Essential oil compositions of wild garlic (*Allium vineale*) growing wild in north Alabama.

RI ^a^	Compound	Percent Composition			
		#1 ^b^	#2	#3	#4
837	2-Furaldehyde	2.2	1.0	1.3	1.3
854	(2*E*)-Hexenal	0.8	2.0	1.9	2.0
856	(3*Z*)-Hexenol	tr ^c^	1.4	tr	tr
902	2,4-Dimethylthiophene	0.8	1.7	2.0	2.2
915	Allyl methyl disulfide	6.1	3.6	2.3	2.3
930	Methyl (*Z*)-1-propenyl disulfide	3.1	1.1	1.3	1.5
939	Methyl (*E*)-1-propenyl disulfide	12.5	3.2	2.6	3.0
958	Benzaldehyde	tr	0.6	4.2	16.4
965	Dimethyl trisulfide	17.4	3.8	4.4	4.3
1079	Diallyl disulfide	6.3	12.2	4.4	5.2
1091	Allyl (*Z*)-1-propenyl disulfide	3.1	4.3	3.4	2.8
1099	Allyl (*E*)-1-propenyl disulfide	11.6	12.5	7.9	8.2
1115	1-Propenyl propyl disulfide ^d,e^	1.4	2.0	1.9	1.7
1123	Methyl methylthiomethyl disulfide	tr	0.5	1.2	0.8
1135	Allyl methyl trisulfide	13.2	9.9	9.9	7.9
1147	4-Methyl-1,2,3-trithiolane ^d^	tr	1.8	1.0	1.6
1149	Methyl propyl trisulfide	tr	1.9	2.7	1.8
1158	Methyl (*Z*)-1-propenyl trisulfide	1.9	0.5	1.4	0.9
1164	Methyl (*E*)-1-propenyl trisulfide	2.7	1.0	1.7	1.5
1211	Dimethyl tetrasulfide	4.0	0.8	1.2	1.1
1284	Allyl methylthiomethyl disulfide ^d^	tr	2.3	2.6	1.9
1291	Diallyl trisulfide	2.8	10.5	7.9	5.3
1302	Allyl (*Z*)-1-propenyl trisulfide	tr	3.0	2.7	2.3
1309	*p*-Vinylguaiacol	5.3	5.2	6.5	5.4
1320	Allyl propyl trisulfide	tr	1.1	2.4	2.2
1344	5-Methyl-1,2,3,4-tetrathiane ^d^	tr	5.5	6.1	4.3
1346	Methyl methylthiomethyl trisulfide	tr	0.7	1.0	1.5
1364	Allyl methyl tetrasulfide	2.4	1.6	2.4	1.8
1483	Allyl methylthiomethyl trisulfide ^d^	tr	0.5	1.2	0.9
1599	Unidentified ^f^	1.6	1.7	4.6	3.3
1623	4-Methyl-1,2,3,5,6-pentathiepane ^d^	tr	0.5	2.0	1.2
1754	Unidentified ^g^	0.6	2.0	4.1	3.2
	Total Identified	97.8	96.2	91.4	93.5
	Sulfur-containing	91.6	90.0	86.1	74.9

^a^ RI = Retention index determined with respect to a homologous series of *n*-alkanes on an HP-5ms column. ^b^ #1–#4 are different essential oil samples. ^c^ tr = trace (<0.05%). ^d^ Identification based on MS only. ^e^ (*Z*)/(*E*)-Isomer not determined. ^f^ MS (m/z): 410 (4%), 326 (3%), 221 (21%), 207 (5%), 129 (81%), 69 (100%), 59 (26%), 45 (32%), 41 (31%). ^g^ MS (m/z): 446 (2%), 405 (2%), 269 (2%), 207 (5%), 129 (71%), 69 (100%), 59 (17%), 45 (27%), 41 (35%).
